# Ventilation in Operating Theatres

**Published:** 1909-10-30

**Authors:** Keith D. Young

**Affiliations:** 17 Southampton Street, Bloomsbury, London, W.C.


### VENTILATION IN OPERATING THEATRES.

Sir,—In Mr. Lockwood's interesting lecture on the methods of attaining asepsis in operation theatres he puts the cubic capacity of a theatre to hold twenty people, in addition to the patient, at 8,000 to 10,000 cubic feet, and recommends that the air should be changed at least six times in the hour. Now, the recognised amount of air which should be supplied to each person in the room per hour is 3,000 ft. For twenty-one persons, therefore, the total amount required per hour is 63,000 ft. Taking the lesser dimension of 8,000 cubic feet, the air would have to be changed nearly eight times per hour; and the larger dimension would require a change of over six times (6.30). The amount of 3,000 cubic feet per head per hour was computed by De Chaumont as the quantity necessary "so to dilute the carbonic acid gas expired by an adult male in repose that the excess of carbonic acid gas in the air over that in the fresh air supplied is maintained at two volumes per 10,000; that limit of perception of closeness of smell" (W. N. Shaw, F.R.S., "Air Currents and the Laws of Ventilation"). And though more recent authorities have recommended a lower standard, Dr. Shaw, in the work quoted, maintains that there is sufficient physical ground for adhering to De Chaumont's figures.

It would seem, therefore, that an accurate mode of arriving at the amount of air necessary for the ventilation of a theatre (or, indeed, of any other room) is by calculating the amount on De Chaumont's method and arranging the apparatus accordingly, rather than by assuming a more or less empirical rule for changing the air so many times per hour.

Another important point raised by Mr. Lockwood is the quality and unit of resistance of the filtering material. It would be useful if some careful experiments could be made from which definite rules could be deduced giving the rate of flow of air through different thicknesses of filtering material. I am, Sir, yours faithfully,

Keith D. Young.

17 Southampton Street, Bloomsbury, London, W.C., October 22, 1909.

